# Diagnostic and treatment barriers to persistent somatic symptoms in primary care – representative survey with physicians

**DOI:** 10.1186/s12875-021-01397-w

**Published:** 2021-04-01

**Authors:** Marco Lehmann, Nadine Janis Pohontsch, Thomas Zimmermann, Martin Scherer, Bernd Löwe

**Affiliations:** 1 Clinic and Outpatients Clinic for Psychosomatic Medicine and Psychotherapy, University Medical Center Hamburg-Eppendorf, Martinistr. 52, 20246 Hamburg, Germany; 2 Department of General Practice and Primary Care, University Medical Center Hamburg-Eppendorf, Hamburg, Germany

**Keywords:** Persistent somatic symptoms, Medically unexplained symptoms, Consultation, Doctor-patient relationship, Primary care, Survey

## Abstract

**Background:**

Many patients consult their primary care physician with persistent somatic symptoms such as pain or sickness. Quite often these consultations and further diagnostic measures yield no medical explanation for the symptoms – patients and physicians are left in uncertainty. In fact, diagnostic and treatment barriers in primary care hinder timely health-care provision for patients suffering from persistent somatic symptoms (PSS). The significance of individual barriers is still unknown. We compare and quantify these barriers from the perspective of primary care physicians and identify subpopulations of primary care physicians who experience particular barriers as most severe.

**Methods:**

We mailed a questionnaire to primary care physicians (PCP) in Germany and asked them which barriers they consider most important. We invited a random sample of 12,004 primary care physicians in eight federal states in Germany. Physicians provided anonymous mailed or online responses. We also mailed a postcard to announce the survey and a mail reminder. Main measures were Likert rating scales of items relating to barriers in the diagnosis and treatment of PSS in primary care. Information on demography and medical practice were also collected.

**Results:**

We analyzed 1719 data sets from 1829 respondents. PCPs showed strongest agreement with statements regarding (1.) their lack of knowledge about treatment guidelines, (2.) their perceptions that patients with PSS would expect symptom relief, (3.) their concern to overlook physical disease in these patients, and (4.) their usage of psychotropic drugs with these patients. More experienced PCPs were better able to cope with the possibility of overlooking physical disease than those less experienced.

**Conclusions:**

The PCPs in our survey answered that the obligation to rule out severe physical disease and the demand to relieve patients from symptoms belong to the most severe barriers for adequate treatment and diagnosis. Moreover, many physicians admitted to not knowing the appropriate treatment guidelines for these patients. Based on our results, raising awareness of guidelines and improving knowledge about the management of persistent somatic symptoms appear to be promising approaches for overcoming the barriers to diagnosis and treatment of persistent somatic symptoms in primary care.

**Trial registration:**

German Clinical Trials Register (Deutschen Register Klinischer Studien, DRKS)

https://www.drks.de/drks_web/setLocale_EN.do

The date the study was registered: October 2^nd^ 2017

The date the first participant was enrolled: February 9^th^ 2018

DRKS-ID: DRKS00012942

**Supplementary Information:**

The online version contains supplementary material available at 10.1186/s12875-021-01397-w.

## Key messages

- PCPs face patient expectations for relief from persistent somatic symptoms
- PCPs admit lack of guideline knowledge of somatoform and functional disorders
- PCPs worry about overlooking physical disease in somatoform patients

## Background

Despite the high prevalence of persistent somatic symptoms (PSS) in the general population and primary care [1, 2], the health-care system offers inadequate help for afflicted patients [3]. Symptoms causing severe impairment have a point prevalence of 22.1% in the general population [1]; in primary care, more than 60% of patients show at least one medically unexplained symptom [2]. *Persistent somatic symptoms* is used today as an umbrella term to describe subjectively distressing somatic complaints irrespective of their etiology, and therefore includes symptoms caused by clear medical pathophysiology, medically unexplained symptoms (MUS), and functional symptoms. Patients with MUS show high symptom burden and costly health-care utilization [4, 5]. Employees with high somatic symptom severity have comparably longer durations of sick leave [6]. Available evidence suggests that primary care physicians (PCPs) sometimes perceive patients with PSS or MUS as a burden [7]. In a Danish study, PCPs reported experiencing almost one third of consultations with patients showing multiple somatic symptoms as burdensome [7].

Reviewing the clinical picture as it presents in primary care consultations, the high strain on patients and PCPs becomes clear. About 40% of patients with medically unexplained symptoms show signs of anxiety, depression, or abridged somatization disorder [8]. Further, Barsky and Borus [9] describe that patients with functional symptoms believe to be seriously ill and present their situation as catastrophic. To this end, patients with somatization disorder are often inclined to undergo physical examination or even surgery despite the medical inexplicability of their symptoms [10]. Furthermore, afflicted patients request explanations for their symptoms from their PCPs. Although there is a multitude of bio-psycho-social explanatory models available for primary care consultation [11], some patients persistently assume a missed severe physical disease could explain their symptoms [12]. The reassuring effect of negative diagnostic results is usually only short-term [13], therefore patients demand frequent and expensive diagnostic procedures [14]. Despite this, symptoms can switch back and forth between medical explicability and inexplicability [15]. In this configuration it is not surprising that Herzog and colleagues found a substantial duration of untreated illness (mean 25.2 years) in 139 patients fulfilling life-time diagnosis of a somatoform disorder, 68% per cent of them reporting persistent pain disorder [3].

Results of a systematic review recently conducted by our team indicate that several barriers impede successful diagnosis, treatment, and management of functional or somatoform disorders in primary care [16]. The barriers emanate from the patient, the PCP, and their interaction, the health-care system, and the concept of somatoform disorder. PCP related barriers further subdivide into communication and consultation behavior, predominance of a biomedical disease model, attitudes towards patients, perception of patient wishes and expectations, knowledge about somatoform and related disorders, and lack of confidence [16]. However, it is unknown which barriers hinder adequate diagnosis and treatment most. We do not know their impact in primary care practice. Knowledge about the strongest barriers could allow for improvements to the health-care system and presumably enable earlier treatment for patients.

## Methods

We investigated a representative sample of PCPs to obtain their views regarding the clinically most important barriers. Our research questions were: (1.) What are the most severe barriers for PCPs in the diagnosis and treatment of patients with PSS? (2.) Which characteristics of the PCP or of primary care practice are associated with these barriers?

A cross-sectional representative anonymous survey was administered with PCPs in eight federal states of Germany from February 9^th^ to May 15^th^, 2018. The survey was registered October 2^nd^, 2017 at the German Clinical Trials Register (DRKS00012942), a primary World Health Organization (WHO) register meeting the requirements of the International Committee of Medical Journal Editors (ICMJE). We obtained ethical approval from the Ethics Committee of the Hamburg Medical Association, Germany, on April 7^th^, 2015 (approval number PV4763). The survey description complies with the Strengthening the Reporting of Observational Studies in Epidemiology (STROBE) checklist for cross-sectional studies (Version 4, 2007).

### Participants

PCPs were randomly sampled (*n* = 12,004). The sampling frame consisted of *N* = 15,389 PCPs, which is the total number of working PCPs from the German federal states of Schleswig–Holstein, Hamburg, North Rhine, Saarland, Brandenburg, Saxony, Mecklenburg-Vorpommern, and Thuringia. The sampling frame and contact information of the PCPs was publicly accessible through registries of associations of statutory health insurance physicians. We sampled proportionally to the total number of primary care physicians working in the respective federal states. The time coverage of the sampling frame was April 2016 to January 2017. PCPs were eligible to participate if they worked as a physician in primary care. We invited both employed and self-employed PCPs. Participation was completely voluntary and anonymous. To comply with common research practice in Germany, we offered no incentive.

### Survey

The survey comprised questions about how strongly the six PCP-related barriers [16] apply in primary care: (1.) attitudes and knowledge of somatoform type problems, (2.) attitude towards patients, (3.) predominance of a biomedical disease model, (4.) perceptions of patient beliefs, wishes and expectations, (5.) communication and consultation behavior, and (6.) lack of confidence. To each of these barrier categories, we allocated three or four items, respectively (supplementary material), totaling at 21 items. These barrier-related items originated from our focus group study [17] and interviews with PCPs and patients with PSS regarding barriers to their diagnosis [18]. Furthermore, three items covering symptom-focused management, the ability to avoid stigmatizing comments, and bio-psycho-social diagnostics were geared to the guideline for functional bodily complaints by the Association of the Scientific Medical Societies in Germany [19]. This guideline comprises practical advice for the diagnosis and therapy of functional bodily symptoms (including persistent somatic symptoms). It originates from the cooperation of more than thirty professional societies and a systematic literature review of more than 3500 articles. Finally, we adapted one item from an earlier survey [20] about whether PCPs would enjoy working with patients with medically unexplained symptoms. All items were discussed and their wording was tightened by the study team. They were then passed on to six researchers with degrees in psychology involved in empirical psychosomatic research and psychometrics to review their wording and ensure their suitability for a scientific survey. The items were further passed on to five PCPs to check their comprehensibility for PCPs and relevance for clinical practice. In the survey, the items were presented as Likert rating scales with six response options ranging from 1: does not apply to 6: applies completely. We included another 21 items regarding personal characteristics of the PCP and their practice. Personal characteristics were gender, age, medical specialty, additional qualification, and years of clinical practice. Practice characteristics distinguished between own practice and practice collaboration, practice location in an urban or a more rural region, and the average number of patients treated in three months.

### Data acquisition

PCPs were invited by mail to participate. They received a post card announcement and two consecutive mailings of the survey package. Data acquisition commenced February 9^th^, 2018 with the announcement one week prior the first mailing of the survey, with the second mailing following two weeks later. Data collection was closed on May 15^th^, 2018. There was also an option for online participation. The invitation to participate consisted of a data safety statement, assurance of anonymous data analysis, and statement that participation was completely voluntary. Informed consent was presumed with the return of completed response forms.

### Statistical analysis

Non-responders and responders were compared regarding gender and medical specialty in the analysis of demographics. Furthermore, the distribution of responders regarding gender, medical specialty, and practice characteristics was compared with official statistics of the population of primary care physicians [21–23]. Next, the mean values of all 21 barrier items were calculated with 95% confidence intervals. Items which show high agreement on average were analyzed further in multiple linear regressions. These regressions tested *R*^2^ as the strength of association between single barrier items (i. e., items with the highest means) and characteristics of the person and the practice as predictors. Qualitative predictors were dummy coded using one reference category, respectively. Multicollinearity between predictors was checked using variance inflation factor.

**Fig. 1:**
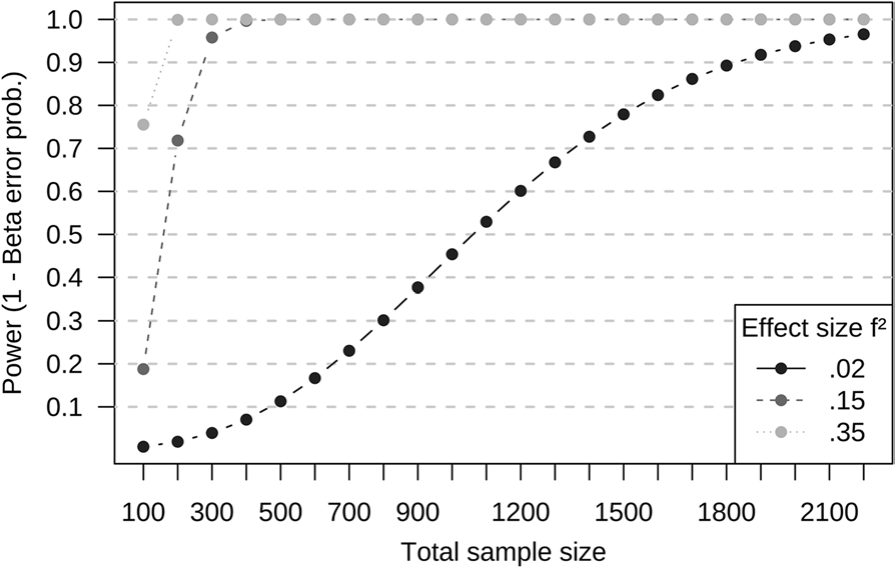
Power analysis for *R*^2^ Tests in a multiple linear regression. Fixed model, number of predictors = 15, α error probability = 0.0025. Calculated using G*Power Version 3.1.9.2

Sample size was planned for linear regression for 20 F-tests of the R-squared coefficient (Bonferroni adjusted α = 0.05 / 20 = 0.0025, 15 predictors). We used effect size specifications *f*^2^ corresponding with Cohen [24]: “small” 0.02, “medium” 0.15, and “large” 0.35. Figure 1 displays the achieved power given a wide range of possible sample sizes and given the different effect sizes. For medium and large effect sizes, power reaches levels beyond 0.90 with less than 300 participants. For small effect sizes, power reaches 0.80 with slightly less than 1600.

## Results

**Fig. 2:**
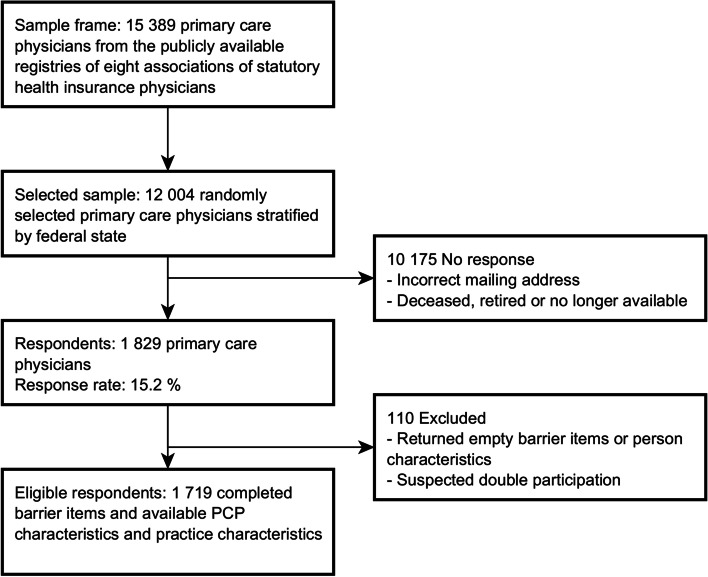
Study flow chart

From our sample of 12,004 PCPs, we obtained responses from a total of 1829 PCPs (15.2%) (Fig. 2). Among the reasons given for non-participation were retirement, death of the PCP, comments that the survey method was inappropriate, and non-specified reasons. Non-participation was not related to the topic of persistent somatic symptoms. Of the 1829 responses, 110 were incomplete or portended double participation. These were omitted from the analysis; as a result, 1719 cases were included.

The distributions of gender and medical specialty of the responders differed from the respective distributions in the sample and the official statistics (Table 1). That is, more women than expected participated. The distribution of medical specialty of the responders only followed the same rank order as found in the sample and in the official statistics. The distribution of practice setting closely followed the distribution in the official statistics. For the number of patients treated per quarter, again, the rank order of the responder distribution followed the order found in the official statistics.

**Table 1:** Personal and practice characteristics of the participating German primary care physicians, *n* = 1719

	Level	Responder	Sample	Official statistics
n		1 719	12 004	54 741 ^a^
Gender (%)	male	755 (44.6)	6 063 (50.5)	30 201 (55.2) ^a^
	female	936 (55.4)	5 940 (49.5)	24 540 (44.8) ^a^
Medical specialty (%)^d^	General medicine	1 134 (67.4)	6 960 (58.0)	34 751 (62.0) ^a^
	Internal medicine	429 (25.5)	3 543 (29.5)	15 417 (27.5) ^a^
	Other or none	77 (4.6)	1 501 (12.5)	4 852 (8.7) ^a^
	General and internal medicine	42 (2.5)	-	1 047 (1.9) ^b^
Years of experience as PCP (%)	0–10 years	454 (27.4)	-	
	11–20 years	540 (32.5)	-	
	21–30 years	454 (27.4)	-	
	> 30 years	211 (12.7)	-	
Practice setting (%)	Own practice	836 (50.1)	-	26 823 (49.0) ^c^
	Collaborative practice	834 (49.9)	-	27 918 (51.0) ^c^
Number of patients (%)	less than 500	53 (3.2)	-	1 642 (3.0) ^c^
	500–1000	654 (39.7)	-	11 879 (21.7) ^c^
	more than 1000	942 (57.2)	-	37 224 (68.0) ^c^
			-	
Practice region (%)	Rural (max. 4 999 Inh.)	321 (19.6)	-	
	Small town (5 000 to 19 999 Inh.)	369 (22.5)	-	
	Medium town (20 000 to 99 999 Inh.)	351 (21.4)	-	
	Large town (above 100 000 Inh.)	599 (36.5)	-	
Additional qualification (%)	Psychotherapy	98 (5.7)	-	
	Basic psychosomatic care	1 163 (67.7)	-	
	other or none	458 (26.6)	-	
Weekly proportion of patients with somatoform disorders (%)	0–10%	711 (51.4)	-	
	11–20%	342 (24.7)	-	
	21–30%	208 (15.0)	-	
	> 30%	123 (8.9)	-	

^a^National Association of Statutory Health Insurance Physicians [21]

^b^German Medical Association [22],

^c^INFAS & National Association of Statutory Health Insurance Physicians [23]

^d^The sum of figures in medical specialty of official statistics (56 067) is slightly higher than the count for all PCPs (54 741), because the source gave counts for physicians according to their title “general medicine” and “others or none”. However, some of those are not working as PCPs, but as specialists

**Fig. 3:**
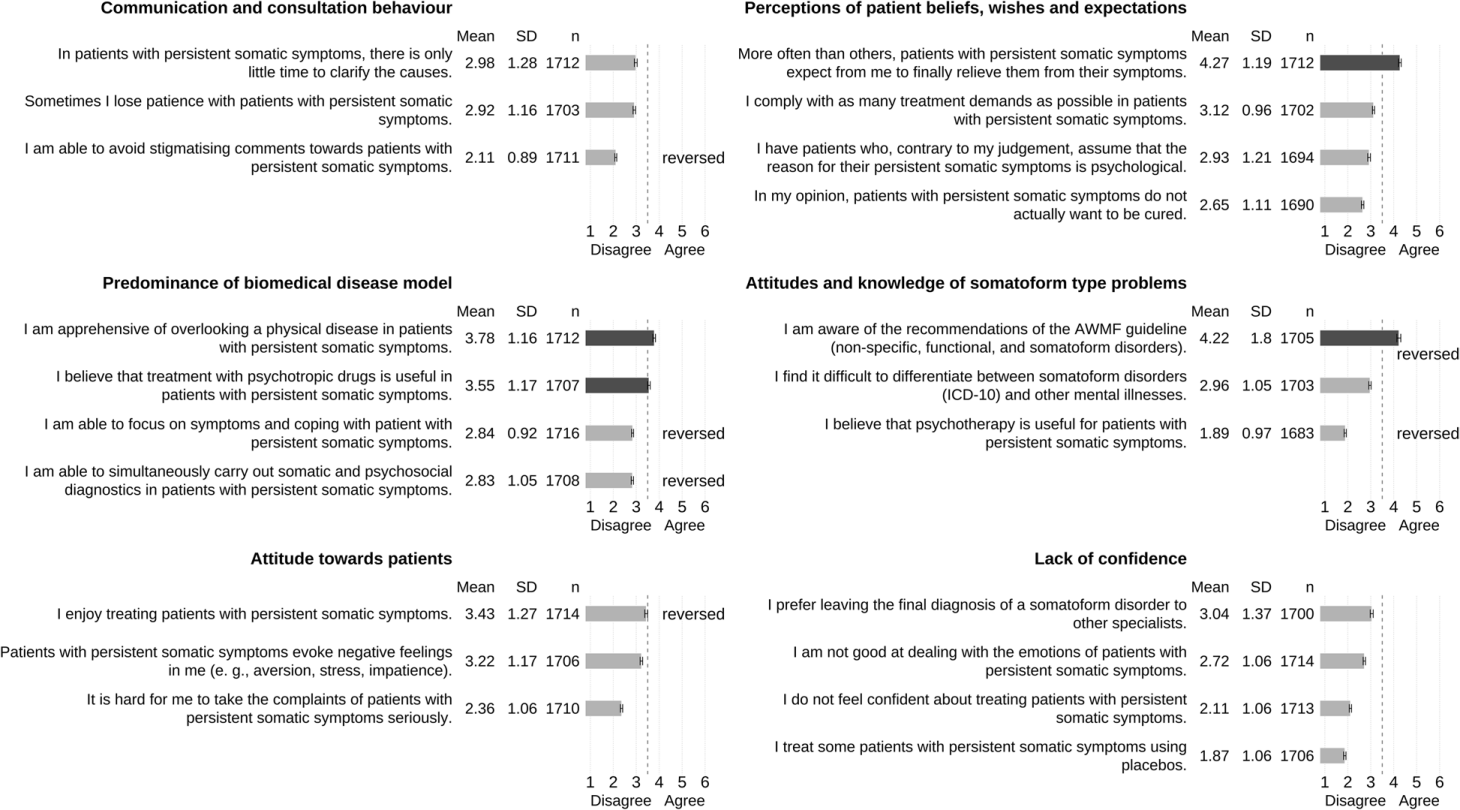
Barriers for Primary Care Physicians in diagnostic and treatment of persistent somatic symptoms, *n* = 1719. Items are grouped by categories according to Murray and colleagues [16]. For six items, the agree-disagree dimension is reversed as indicated

Figure 3 shows unconditional means and 95% confidence intervals for the 21 items we subsumed under the six PCP related barriers. For a consistent display, we reversed six items as indicated so that for all items, higher rating reflects a stronger barrier. Most of the employed items score in the left part of the scale indicating disagreement and, due to the large sample size, show narrow confidence limits. However, the bars of four items indicated in a different color cross the middle line of the scale, indicating agreement. The PCPs most strongly agreed with how they perceive the patients, in that patients with persistent somatic symptoms expect PCPs to relieve their symptoms. In the item showing the next highest agreement, PCPs admitted a lack of knowledge relating to somatoform type complaints due to unawareness of the pertinent guidelines for the management of non-specific, functional and somatoform disorders. Rank three and four of the items with highest agreement pertain to the barrier of a predominantly biomedical disease model. That is, the PCPs reported to be apprehensive of overlooking physical disease and expressed the belief that treatment with psychotropic drugs would be useful in patients with PSS. In contrast to the items with high agreement, there were also items which were not evaluated as problematic by the PCPs. To this end, PCPs indicated their ability to avoid stigmatizing comments with patients, that they would not use placebo treatment with patients, that they are aware psychotherapy is useful for patients with PSS, and that they are confident in treating these patients.

The multiple regression analysis aimed to use the characteristics of the PCP and her or his practice to explain the variance of the barrier items with the highest agreement. The items were the use of psychotropic drugs, the concern to overlook physical disease, the perception that patients expect symptom relief, and the lack of guideline knowledge. These items were used as regression criteria (Table 2). Predictors were characteristics of the person (i.e., years of experience as PCP and gender) and characteristics of the practice (i.e., practice setting, number of patients, and practice region). The generalized variance inflation factor for all predictors was at maximum VIF = 1.09. We assessed that multicollinearity was not an issue in this data set.

**Table 2:** Multiple linear regression of diagnostic and treatment outcomes on PCP and practice characteristics

	Outcome			
	Use of psychotropic drugs	Concern to overlook physical disease	Relief from symptom burden	Lack of guideline knowledge
	B (CI 95%)	B (CI 95%)	B (CI 95%)	B (CI 95%)
Years of experience as PCP				
11–20	0.06 (-0.09, 0.22)	-0.27^***^ (-0.42, -0.12)	0.03 (-0.13, 0.19)	0.13 (-0.10, 0.37)
21–30	-0.02 (-0.18, 0.14)	-0.25^**^ (-0.41, -0.09)	-0.06 (-0.23, 0.10)	0.21 (-0.04, 0.46)
> 30	0.08 (-0.12, 0.28)	-0.44^***^ (-0.64, -0.24)	-0.05 (-0.25, 0.16)	0.001 (-0.31, 0.32)
Gender female	0.05 (-0.07, 0.17)	0.04 (-0.08, 0.16)	-0.04 (-0.16, 0.09)	-0.30^***^ (-0.49, -0.12)
Collaborative practice	0.06 (-0.07, 0.18)	-0.01 (-0.13, 0.11)	0.07 (-0.06, 0.19)	0.06 (-0.13, 0.25)
Practice region				
Small town (5 000–19 999 Inh.)	-0.12 (-0.30, 0.06)	-0.13 (-0.31, 0.05)	0.02 (-0.17, 0.21)	-0.11 (-0.39, 0.17)
Medium town (20 000–99 999 Inh.)	0.02 (-0.16, 0.20)	-0.09 (-0.27, 0.09)	0.06 (-0.12, 0.25)	0.08 (-0.21, 0.37)
Large town (> 100 000 Inh.)	0.02 (-0.15, 0.19)	-0.19^*^ (-0.36, -0.03)	-0.04 (-0.21, 0.13)	-0.09 (-0.35, 0.17)
Number of patients				
500–1000	-0.02 (-0.38, 0.34)	-0.24 (-0.60, 0.11)	0.07 (-0.30, 0.44)	0.26 (-0.30, 0.82)
1001–1500	0.14 (-0.22, 0.50)	-0.15 (-0.51, 0.21)	0.15 (-0.22, 0.52)	0.31 (-0.25, 0.87)
> 1500	0.29 (-0.09, 0.66)	-0.32 (-0.69, 0.05)	0.07 (-0.32, 0.45)	0.02 (-0.57, 0.60)
Constant	3.38^***^ (2.99, 3.78)	4.29^***^ (3.90, 4.69)	4.18^***^ (3.77, 4.59)	4.08^***^ (3.47, 4.70)
Observations	1,492	1,494	1,497	1,489
*R*^2^	0.01	0.02	0.004	0.01
Adjusted *R*^2^	0.01	0.02	-0.003	0.01
Residual Std. Error	1.15 (df = 1480)	1.14 (df = 1482)	1.19 (df = 1485)	1.80 (df = 1477)
F Statistic	1.73 (df = 11; 1480)	3.42^***^ (df = 11; 1482)	0.58 (df = 11; 1485)	1.99^*^ (df = 11; 1477)

Note:^*^p < 0.05; ^**^p < 0.005; ^***^p < 0.0025 (Bonferroni adjusted alpha level for 20 statistical tests)

Regarding the multiple regression coefficient R-squared, only the concern to overlook physical disease was significant at the adjusted alpha level of α = 0.0025 and with an explained variance of 2%. Inspection of the comprised coefficients for single predictors showed that PCPs with more years of experience were less concerned to overlook physical disease, as compared to PCPs with 0–10 years of experience. The lack of guideline knowledge only reached significance if alpha adjustment was not considered. The strongest single predictor for this criterion was gender: being female was associated with better guideline knowledge. Apart from the regression, the bivariate association between guideline knowledge and gender yielded the same result that females had better knowledge of guidelines than males (M_female_ = 4.08, M_male_ = 4.39, t = 3.6, df = 1618, *p* < 0.0004). Replication of the regression calculations with imputed predictor variables yielded the same pattern of R-squared and regression coefficients.

## Conclusions

Our results identified the most relevant barriers for the diagnosis and treatment of patients with PSS in primary care. The survey items showing the highest agreement exemplify these barriers: (1.) The PCPs perceived patients’ expectations that they would remedy the PSS. (2.) They also recognized a lack of knowledge about the pertinent diagnostic and treatment guideline about somatoform and functional disorders. (3.) They were worried of overlooking physical disease and (4.) they appraised psychotropic drugs as useful for PSS treatment. The PCPs agreed least with (1.) the disregard of psychotherapy as helpful, (2.) the use of stigmatizing comments in consultation, (3.) the use of placebo treatment with patients, and (4.) the lack of confidence for the treatment of patients with PSS. The four items with the highest agreement were associated with only few characteristics of the PCP or his or her practice, as our regression analysis showed. Evidently, PCPs with less than 10 years of experience in general practice were more concerned about overlooking physical disease than more experienced PCPs. Lack of guideline knowledge was associated with male gender. Interestingly, notwithstanding our large sample size, no other regression coefficients showed statistical significance.

This pattern of results, that is, the four items with the highest agreement and their lack of associations with personal or practice characteristics, may suggest that diagnostic and treatment barriers for patients with PSS apply irrespective thereof. According to our data, the concern to overlook physical disease [25] and the patient expectation of symptom relief are the two demands that PCPs face. Furthermore, many patients with PSS request explanations for symptoms which have no satisfactory bio-medical explanation [26]. Trying medication may seem reasonable and PCPs often prescribe medicine in response to the patients’ symptom elaboration [27]. However, there is scarce evidence that pharmacological intervention improves medically unexplained symptoms [28], which are characteristic of many patients with PSS. Furthermore, our focus groups with PCPs suggest that the obligation to rule out physical disease is so strong that it cannot be bypassed with short-term reassurance or medication [29]. Simultaneously, patients with PSS often express hints of psychosocial symptom burden [30]. This burden is often not acknowledged by PCPs [31], although it may also figure in a tenable explanation of the symptoms. Nevertheless, whether or not psychosocial burden is present, overlooking physical disease in a patient with PSS is still possible, explaining the PCPs’ ambivalence regarding a possible medical diagnosis [32]. Moreover, the decision whether medically explicable or not is never conclusive and can be swayed in both directions with further diagnostics [15]. The situation with patients with PSS in primary care described above lets us understand the negative emotions that many PCPs bring to the management of these patients [33] and, possibly, why PCPs prescribe psychotropic drugs to them [27].

One of the essentials of guideline oriented management of functional disorders is to take the patients’ hints of psycho-social burden in the consultation more seriously [34], perhaps even if they are unwilling to openly reveal them [35]. For example, the BATHE procedure provides specific guidance for PCPs to consider stress and affect in their consultations [36] and the 4DSQ can be particularly useful to identify somatization associated with PSS [37]. Furthermore, in comparison with the former somatoform disorder, the diagnostic innovations of somatic symptom disorder (Diagnostic and Statistical Manual of Mental Disorders 5, DSM-5) and bodily distress disorder (International Statistical Classification of Diseases and Related Health Problems 11, ICD-11) may promote stronger consideration of psycho-social burden in primary care. For example, the B-criteria of somatic symptom disorder ask for excessive symptom-related concern, anxiety, and behavior. So, in order to assign the diagnosis, the clinician must assess these psychological symptoms, irrespective of whether the physical symptoms are medically explained or not, or use a screening instrument like the SSD-12 to do so [38–40]. In fact, medically explained and unexplained symptoms both entail subjective impairment and change in lifestyle [15]. PCPs might be quite open-minded to these diagnostic innovations, as our focus group study suggests [29], therefore primary care consultation could address these issues with the help of appropriate management and psychological treatment strategies. Admission of a lack of guideline knowledge, as evident in our data, and the willingness to learn about how to identify medically unexplained symptoms [41] are steps in the right direction. To embrace the bio-psycho-social model suggested by George L. Engel [42] would further benefit patients with PSS, as Rasmussen and colleagues have previously expressed for patients with medically unexplained symptoms in particular [32].

A strength of our survey is the inclusion of a large representative sample. We therefore consider our results to be unbiased regarding the population of PCPs in Germany. The design was sensitive for small to medium effect sizes as an a priori power analysis revealed. The anonymous survey allowed PCPs to be as open as possible. However, there is the possibility of social desirability bias with certain items, for example, whether PCPs can avoid stigmatizing comments in clinical consultation. Items were selected with regard to their content and fit to the barriers identified in our previous systematic review, earlier research findings, psychological expert judgment, and the expert judgment by PCPs. Thereby, we ensured maximum content validity of each item. However, to further enhance the understanding of the items, a case vignette at the outset could have helped the PCPs to consider more of their personal experiences in their responses. A limitation is the response rate of about 15%. Although this rate can be considered comparably low, we do not suspect loss of generalizability due to the random sampling procedure. Moreover, in the German health-care system, rather low response rates are common [43, 44] and even a considerable incentive might not have raised the rate satisfactorily [45]. It could have been advantageous if the invitation to participate had been promoted by a national PCP body; however, this could have interfered with our declaration that there is no particular interest in the results and, therefore, could have introduced bias.

Patients with persistent somatic symptoms will continue to demand symptom relief from their PCPs and the exclusion of physical illnesses will continue to be a central concern for PCPs, too. How to prioritize efforts to amend barriers may be obvious from our data – work against the four strongest barriers – but it may be difficult to implement: Support PCPs to manage the demand for symptom relief and give clear advice and legal protection in the exclusion of a physical illness in patients with persistent somatic symptoms. Furthermore, disseminate guideline knowledge even more and knowledge about the role of medication in patient management and therapy. Taking up the interest of PCPs to enhance management of patients with PSS, more research could tackle the long standing issue of how to better disseminate guideline knowledge [46] and the diagnostic innovations of somatic symptom disorder (DSM-5) and bodily distress disorder (ICD-11).

## Supplementary Information

**Additional file 1**

## Data Availability

The datasets used and/or analyzed during the current study are available from the corresponding author on reasonable request.

## Abbreviations

DRKS: German Clinical Trial Register
DSM-5: Diagnostic and Statistical Manual of Mental Disorders
ICD-11: International Statistical Classification of Diseases and Related Health Problems
ICMJE: International Commitee of Medical Journal Editors
MUS: Medically unexplained symptoms
PCP: Primary Care Physician
PSS: Persistent somatic symptoms
STROBE: Strengthening the Reporting of Observational Studies in Epidemiology
VIF: Variance Inflation Factor
WHO: World Health Organization
