# Supplementary material for: Diagnostic and treatment barriers to persistent somatic symptoms in primary care – representative survey with physicians
Source: BMC Fam Pract. 2021 Apr 1;22:60. doi: 10.1186/s12875-021-01397-w (PMC8017612; doi:10.1186/s12875-021-01397-w)
Supplement: Supplementary file 1 — Additional file 1 [file 12875_2021_1397_MOESM1_ESM.docx]

# Diagnostic and treatment barriers to persistent somatic symptoms in primary care – representative survey with physicians

Marco Lehmann^a^, Nadine Janis Pohontsch^b^, Thomas Zimmermann^b^, Martin Scherer^b^, Bernd Löwe^a^

^a^Clinic and Outpatients Clinic for Psychosomatic Medicine and Psychotherapy, University Medical Center Hamburg-Eppendorf, Hamburg, Germany

^b^Department of General Practice / Primary Care, Center for Psychosocial Medicine, University Medical Center Hamburg-Eppendorf, Hamburg, Germany

# Supplementary material. Barrier-related items

| **Barrier category** | **Original item (German)** | **Item translation** |
| --- | --- | --- |
| Attitudes and knowledge of somatoform type problems | Ich halte (psycho-) therapeutische Maßnahmen (wie z.B. Einzelpsychotherapie, Gruppenpsychotherapie, Entspannungsverfahren und Psychoedukation) für Patient/innen mit anhaltenden, unklaren und belastenden Körperbeschwerden für sinnvoll. | I believe that psychotherapy is useful for patients with persistent somatic symptoms. |
|  | Ich kenne die Empfehlungen der AWMF-Leitlinie (Nicht-spezifische, funktionelle und somatoforme Körperbeschwerden). | I am aware of the recommendations of the AWMF guideline (non-specific, functional, and somatoform disorders). |
|  | Die Abgrenzung somatoformer Störungen (ICD-10) zu anderen psychischen Störungen fällt mir schwer. | I find it difficult to differentiate between somatoform disorders (ICD-10) and other mental illnesses. |
| Attitude towards patients | Ich behandle Patient/innen mit anhaltenden, unklaren und belastenden Körperbeschwerden gerne. | I enjoy treating patients with persistent somatic symptoms. |
|  | Patient/innen mit anhaltenden, unklaren und belastenden Körperbeschwerden lösen bei mir negative Gefühle aus (z.B. Aversion, Stress, Ungeduld). | Patients with persistent somatic symptoms evoke negative feelings in me (e. g., aversion, stress, impatience). |
|  | Es fällt mir schwer die Beschwerden der Patient/innen mit anhaltenden, unklaren und belastenden Körperbeschwerden ernst zu nehmen. | It is hard for me to take the complaints of patients with persistent somatic symptoms seriously. |
| Predominance of biomedical disease model | Es gelingt mir bei Patient/innen mit anhaltenden, unklaren und belastenden Körperbeschwerden symptom- und bewältigungsorientiert zu arbeiten. | I am able to focus on symptoms and coping with patient with persistent somatic symptoms. |
|  | Ich halte eine Behandlung mit Psychopharmaka bei Patient/innen mit anhaltenden, unklaren und belastenden Körperbeschwerden für sinnvoll. | I believe that treatment with psychotropic drugs is useful in patients with persistent somatic symptoms. |
|  | Bei Patient/innen mit anhaltenden, unklaren und belastenden Körperbeschwerden habe ich die Befürchtung eine organische Erkrankung zu übersehen. | I am apprehensive of overlooking a physical disease in patients with persistent somatic symptoms. |
|  | Es gelingt mir bei Patient/innen mit anhaltenden, unklaren und belastenden Körperbeschwerden eine gleichzeitige somatische und psychosoziale Diagnostik durchzuführen. | I am able to simultaneously carry out somatic and psychosocial diagnostics in patients with persistent somatic symptoms. |
| Perceptions of patient beliefs, wishes and expectations | Patient/innen mit anhaltenden, unklaren und belastenden Körperbeschwerden erfülle ich möglichst viele ihrer Behandlungswünsche. | I comply with as many treatment demands as possible in patients with persistent somatic symptoms. |
|  | Patient/innen mit anhaltenden, unklaren und belastenden Körperbeschwerden konfrontieren mich häufiger als andere Patienten/innen mit der Erwartung, dass ich sie endlich von ihren Symptomen befreie. | More often than others, patients with persistent somatic symptoms expect from me to finally relieve them from their symptoms. |
|  | Ich habe Patient/innen, die entgegen meiner Einschätzung von einer psychischen Ursache ihrer anhaltenden, unklaren und belastenden Körperbeschwerden ausgehen. | I have patients who, contrary to my judgement, assume that the reason for their persistent somatic symptoms is psychological. |
|  | Patient/innen mit anhaltenden, unklaren und belastenden Körperbeschwerden wollen meines Erachtens eigentlich nicht geheilt werden (sekundärer Krankheitsgewinn). | In my opinion, patients with persistent somatic symptoms do not actually want to be cured. |
| Communication and consultation behaviour | Es gelingt mir bei Patient/innen mit anhaltenden, unklaren und belastenden Körperbeschwerden stigmatisierende Kommentare zu vermeiden. | I am able to avoid stigmatising comments towards patients with persistent somatic symptoms. |
|  | Bei Patient/innen mit anhaltenden, unklaren und belastenden Körperbeschwerden bleibt mir wenig Zeit für eine Ursachenklärung. | In patients with persistent somatic symptoms, there is only little time to clarify the causes. |
|  | Manchmal verliere ich die Geduld bei Patient/innen mit anhaltenden, unklaren und belastenden Körperbeschwerden. | Sometimes I lose patience with patients with persistent somatic symptoms. |
| Lack of confidence | Ich kann mit den Emotionen von Patient/innen mit anhaltenden, unklaren und belastenden Körperbeschwerden nicht gut umgehen. | I am not good at dealing with the emotions of patients with persistent somatic symptoms. |
|  | Ich traue mir die Behandlung von Patient/innen mit anhaltenden, unklaren und belastenden Körperbeschwerden nicht zu. | I do not feel confident about treating patients with persistent somatic symptoms. |
|  | Die endgültige Diagnose einer somatoformen Störung überlasse ich lieber anderen Fachärzten/innen. | I prefer leaving the final diagnosis of a somatoform disorder to other specialists. |
|  | Ich führe bei Patient/innen mit anhaltenden, unklaren und belastenden Körperbeschwerden Placebo-Behandlungen durch. | I treat some patients with persistent somatic symptoms using placebos. |
